# The Use of Ripple Effect Mapping to Understand Successes of the SC Pregnancy Assistance Fund: A Participatory Evaluation Approach

**DOI:** 10.1007/s10995-020-02902-w

**Published:** 2020-03-27

**Authors:** Lauren M. Workman, Jennifer S. Browder

**Affiliations:** 1 Core for Applied Research and Evaluation, Arnold School of Public Health, University of South Carolina, 220 Stoneridge Drive, Suite 103, Columbia, SC 29210 USA; 2 Department of Health Services Policy and Management, Arnold School of Public Health, University of South Carolina, 915 Greene Street, Discovery Building, Suite 358, Columbia, SC 29208 USA; 3 Arnold School of Public Health, South Carolina Rural and Minority Health Research Center, University of South Carolina, 220 Stoneridge Drive, Suite 204, Columbia, SC 29210 USA

**Keywords:** Ripple effect mapping, Program evaluation, Community, Teen pregnancy, Collaboration

## Abstract

**Objectives:**

The South Carolina Pregnancy Assistance Fund (SCPAF) funded four counties to increase the amount, quality, and awareness of services for young parents; increase educational attainment among expectant and parenting youth; reduce the number of repeat teen pregnancies among youth; and improve parenting skills. The purpose of this paper is twofold: (1) to describe our application of the Ripple Effect Mapping (REM) technique as an innovative evaluation strategy to gather perspectives from SCPAF stakeholders and (2) to share key findings generated by participants in REM sessions on the perceived success of local SCPAF community collaboratives.

**Methods:**

REM, an innovative evaluation strategy, was used to gather perspectives from SCPAF stakeholders. Five REM sessions were conducted with 52 participants. REM sessions included partner interviews and collective development of visual maps to illustrate stakeholder perspectives of program successes. Visual maps, as well as transcripts of discussions, were analyzed using an inductive approach.

**Results:**

Stakeholders reported that the connections to resources, supports, and services provided through SCPAF had the potential to alter the life trajectories of expectant and parenting teens (EPT). Stakeholders also described that SCPAF fostered growth in collaboration among partners and reduced duplication of services in funded communities

**Conclusions for Practice:**

This paper describes how an innovative evaluation strategy was used to provide a space for stakeholders to dialogue, synthesize their experiences, and construct a collective narrative of key program successes. This paper also illustrates how such approaches can be applied to complex community initiatives.

## Significance

*What is already known on this subject?* Supporting EPT requires comprehensive, community-based, wraparound services from a variety of organizations. However, evaluating community collaboratives to support EPT is challenging because they are complex, multifaceted, and continually evolving. Innovative methods are needed to ensure evaluations capture stakeholder perspectives on a wide range of program outcomes and the context in which they are situated.

*What this study adds?* This study provides an example of how we used an innovative evaluation technique, REM, to understand stakeholder perspectives on the successes of the SCPAF. Additionally, this study describes findings from our evaluation to illustrate how community collaboratives, such as SCPAF, can support EPT.

## Objectives

Although the teen pregnancy rate in the United States has reached historic lows, 2017 data indicate that pregnancies among teens ages 15–19 continue to occur at a rate of 18.8 per 1000 births (Martin et al. 2018). Teenage pregnancy is associated with significant health, social, and economic consequences. For example, teen pregnancy and birth contribute significantly to school dropout rates among females (Kane et al. 2013). Consequently, teenage parents are also more likely to have limited employment opportunities and live in poverty. An estimated 48% of all mothers ages 15–19 live below the poverty line (National Campaign to Prevent Teen and Unplanned Pregnancy 2012).

Given these consequences, expectant and parenting teens (EPT) are a particularly vulnerable population. They have complex needs and thus could benefit from a comprehensive array of services and resources to help them parent effectively and work toward becoming self-sufficient adults. Moreover, the existing population of EPT needs support to promote the health and wellbeing of their families. For example, teen mothers have expressed the need for improved health care; social supports; and services including child care, parenting classes, and peer support (Dumas and Terrell 2017).

However, one program is not likely to address all needs. Having a variety of community programs and resources available to support EPT can integrate care and increase the likelihood of positive outcomes for both the mother and the child (Erickson 2017; Singh et al. 2015). Fostering greater connections between partners and providers and providing care coordination have been attributed to reduced duplication of services, reduced cost of care, and improved care for children and their families (Hassett and Austin 2014).

The South Carolina Pregnancy Assistance Fund (SCPAF) was funded in 2013 through a U.S. Department of Health and Human Services Office of Adolescent Health grant. The overall aim of the Pregnancy Assistance Fund (PAF) grant is to improve the health, educational, social, and economic outcomes of EPT and their families (U.S. Department of Health and Human Services, Office of Adolescent Health 2016). The SCPAF assists EPT ages 15–24 in four communities, which were funded to increase the amount, quality, and awareness of services for young parents; increase educational attainment; reduce the number of repeat teen pregnancies among youth; and improve parenting skills. A state leadership team, led by the Children’s Trust of South Carolina and Fact Forward (formerly the South Carolina Campaign to Prevent Teen Pregnancy), manages community grantees and coordinates technical assistance.

The SCPAF focuses on developing partnerships among youth-serving agencies to create a system of support for EPT, but the state leadership team took a flexible approach to implementation. Hence, community grantees were given autonomy to build upon existing partnerships and capacities. Each community has a lead agency that works to enhance collaboration among partners to provide an array of services. Services include resources for parenting and relationship skills, connections to health care and child care, home visits, mentoring, and job training. Partners in communities across the state include schools; fatherhood organizations; family support agencies; health care providers; vocational training programs; health departments; and local United Way, Salvation Army, and Nurse Family Partnership offices. Although each SCPAF community has its own unique approach, core characteristics include the engagement of a wide range of partners and regular meetings to facilitate collaboration.

The development of collaborative community partnerships is an essential component to community-based programs to support EPT (Butterfoss and Kegler 2002; Goldberg et al. 2011). However, evaluating the complex community-based programs is challenging because it requires coordination of multiple partners and the capacity to monitor initiatives within environments that regularly grow and change. Evaluating how systems change interventions contribute to community transformation is also challenging because such change is large scale and continuously evolving (Kania and Kramer 2011; Wolff et al. 2017). New and innovative evaluation techniques are needed to evaluate complex community collaborations, including evaluating how they are implemented and the results those collaborations produce.

The SCPAF has been evaluated using social network analysis to understand the size, cohesion, and capacity of partnerships (Radcliff et al. 2018). Although this evaluation Radcliffe and colleagues conducted provided essential information on the composition of partnerships, more data on the benefits and effects of those partnerships would enhance our understanding of SCPAF. Therefore, this study was developed to shed further light on the initiative. Ripple Effect Mapping (REM), an innovative evaluation technique, was employed to gather rich, detailed data from stakeholders. The purpose of this paper is (1) to describe stakeholders’ perspectives on the success of SCPAF and (2) to provide an example of an innovative evaluation technique that can be utilized for complex community initiatives.

## Methods

The REM technique is a qualitative evaluation method that aims to engage program stakeholders in describing and visually mapping their perceptions of the effects of a program or complex initiative (Chazdon et al. 2017; Kollock et al. 2012; Olfert et al. 2018). REM has been used to evaluate a variety of programs including community-based health promotion programs (Washburn et al. 2018), child care initiatives (Alviz and Durden 2013), and youth-serving programs (Baker and Johannes 2013).

The REM technique allows stakeholders to synthesize their experiences in real time; explain different perspectives; and reflect together to develop a collective description of program successes (i.e., a “shared narrative”). The REM technique is informed by appreciative inquiry, an orientation toward describing the most positive aspects of an initiative so that the best and most useful aspects of the program can be disseminated, rather than focusing on deficits or what else is still needed (Preskill and Catsambas 2006; Watkins and Cooperrider 2000). However, negative ideas are not excluded from the conversation, and neither are discussions about the unintended or unanticipated impacts.

To evaluate the impacts of SCPAF, we worked with state and community partners to organize five REM sessions. One session was conducted with the state leadership team and the additional four were conducted with funded communities. Each community’s lead agency was asked to invite up to 15 representatives from partner organizations that were involved in some aspect of planning, implementing, or delivering SCPAF activities. The number of participants in each session ranged from 7 to 15; a total of 52 individuals participated across all sessions. SCPAF collaboratives vary in size, so some sessions had a greater number of participants than others had; however, each session did have representation from key stakeholders. A letter of invitation describing the session’s purpose, participants’ rights, and protection of confidentiality was provided to all participants. The University of South Carolina Institutional Review Board approved this study.

Sessions were co-led by a facilitator and a note taker. The “in-depth” rippling approach was used to design our session (Hansen 2015; Chazdon et al. 2017, pp. 27–30). The in-depth REM approach distinguishes three key steps: (1) partner interviews, (2) report out and group mapping, and (3) discussion and reflection. After the facilitator introduced the format for the session, she asked participants to break into pairs and interview their partner using a provided list of questions. Seven questions were developed based on previous REM questions created by Sero et al. (2016). Questions included *What has been the most helpful part of the SCPAF program?, How did the SCPAF program contribute to your community?,* and *What changes resulting from SCPAF are you most proud of?* These interviews were used to set the stage for the group mapping session.

**Fig. 1:**
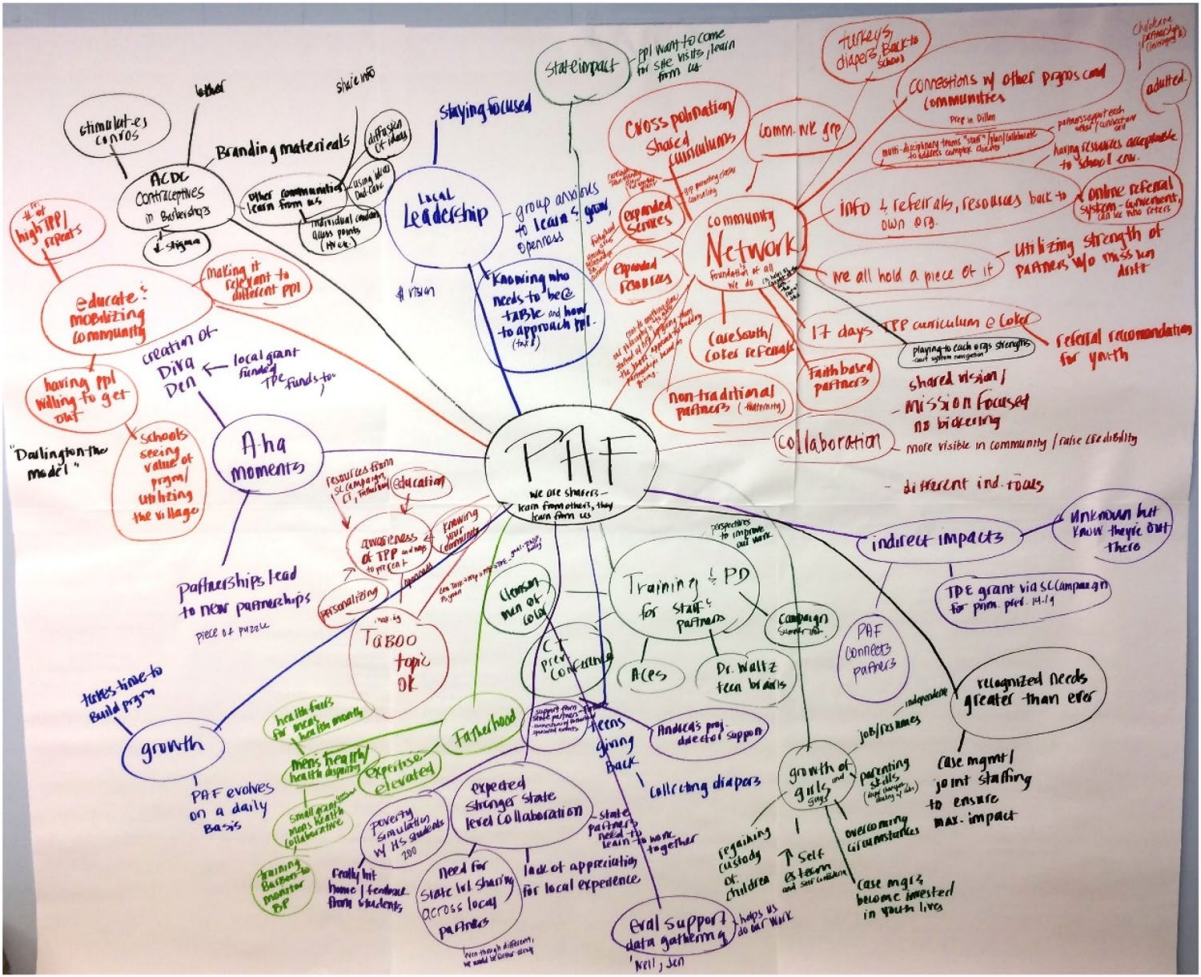
Ripple effect map

Following the interviews, the group reassembled for the mapping session. REMs are built on the concept of a mind map, which is an “image centred, radial diagram that represents semantic or other connections between portions of learned material hierarchically” (Eppler 2006, p. 203). REM calls for hand drawing ideas named by participants in a way that represents connections between those ideas (Eppler 2006). Participants were asked to report what they learned from one another in interviews in a round robin fashion. For example, the facilitator asked participants to “please tell me one thing that you and your partner discussed.” As participants shared their ideas, the facilitator wrote them on a large paper mounted on a wall (Fig. 1). The facilitator probed to gather details about the ideas shared, and other participants were invited to elaborate or contribute additional perspectives. Participants continued to share new topics until they had nothing else to share. Each idea was added onto the paper as a new “ripple.” The facilitator closed the session by asking participants if anything was missed or needed clarification. On average, the sessions lasted 90 minutes to 2 hours. Notes were taken throughout the session, and all sessions were recorded and transcribed for analysis.

**Fig. 2:**
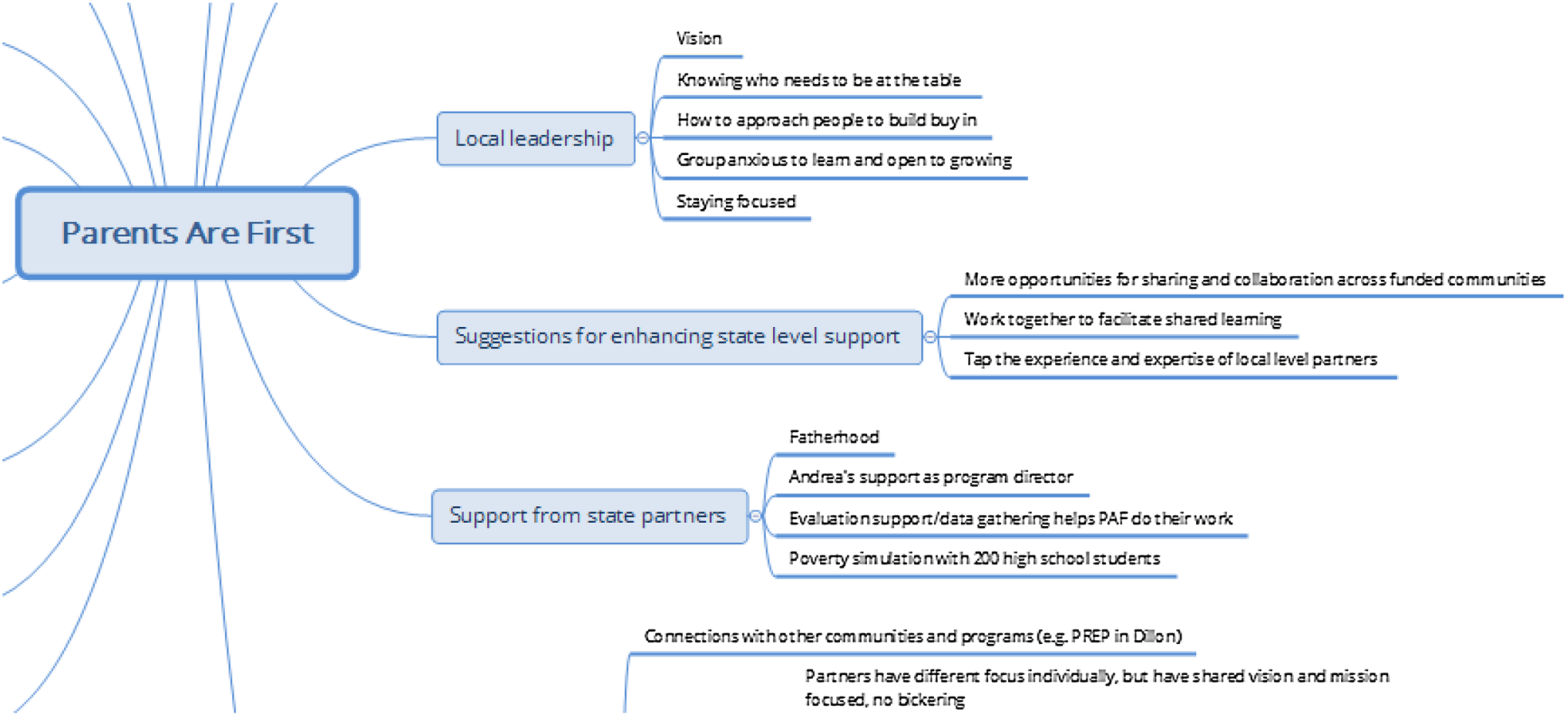
Portion of ripple effect map generated with XMind software

Following data collection, each community map was recorded into mind mapping software (XMind Ltd. 2016; Fig. 2). Mind map nodes were downloaded into Excel and used to develop a coding scheme. We then coded data from transcripts of discussions using NVivo v. 11, a qualitative data analysis software (QSR International Pty Ltd. 2012).

We used an inductive approach to analyze data. Inductive analytical approaches allow for concepts or themes to emerge from raw data, rather than using prior assumptions or theory to guide the analysis (Strauss and Corbin 1998; Thomas 2006). Our analysis was guided by the constant comparison technique, which calls for repeated review of data to allow key themes to emerge (Glaser and Strauss 1967). As our analysis identified emergent themes, some concepts were synthesized into overall themes. These key themes are presented in the results section. We used member checking as a strategy to improve the credibility of our findings (Lincoln and Guba 1985). Accordingly, participants were provided with summary reports and asked for their feedback to verify interpretations.

## Results

Our analysis identified several themes among stakeholders’ perspectives of the successes of the SCPAF. The most notable was their perception that SCPAF could alter the life trajectories of expectant and parenting teens through connections to resources, supports, and services. In addition, stakeholders perceived that SCPAF enhanced accessibility, reduced duplication of services, and improved collaboration among partner agencies.

### SCPAF Connects Youth to Critical Resources

Partners reported observing tangible changes in the youth they engaged in SCPAF. For example, partners explained that youth are getting the support they need and that the resources and services from multiple community agencies have the potential to improve their life trajectories. One community partner explained the value of the partnership in making a collective impact:

One of the things I’ve learned through this partnership is that it is very difficult for one case manager or agency to make an impact…. We are working together to help clients…and the team approach to dealing with an individual situation is very different and helpful…. It’s a lot easier when you have more than one agency to help a young family or mom get to the next step…and that doesn’t happen when you aren’t in a partnership type grant. If a person is affected by more than one agency…the trajectory of their and their child’s life is greatly impacted.

The SCPAF stakeholders described that the initiative improved youths’ access to health care, educational attainment, access to job training, parenting and relationships, access to child care, and connections to counseling. One participant explained how the program supports EPT in completing their education: “Seeing the effectiveness of the program and seeing the difference it makes in students’ lives…. The increase in pride…because of educational attainment…. They recognize because of the support from [the program], ‘I can do this…. I’ve had a baby, but I can still graduate high school.’”

Other important aspects of SCPAF described by partners included the emphasis on programming geared toward fathers, peer support facilitated by group meetings in some communities, and home visits. One stakeholder explained the value of home visits in understanding EPT’s home and family environments: “Home visits are just priceless…to really understand where these students are coming from. They can tell you a story all day long, but until you walk across that threshold or drive in that driveway, sometimes you can’t understand.” REM session participants explained that they anticipated access to these resources and services would have an eventual influence on breaking cycles of poverty and teen pregnancy.

Some SCPAF services and resources have effected other outcomes, as well. For example, some partners perceived that parenting and relationship education provided through SCPAF improved the communication skills of EPT. Participants postulated that this has enhanced the ability of EPT to access employment because they are better able to communicate in job interviews. As a result, staff expect them to be more likely to obtain employment.

### SCPAF Ensures Access to and Coordination of Services but also Reduces Duplication

Community partners shared that the connections and relationships facilitated by SCPAF helped to ensure that EPT connected to needed resources and reduced duplication of services. For example, one person explained that the collaboration has led to “streamlining the process” of connecting teens to vital resources including childcare vouchers and birth control. S/he explained:

We identified issues or barriers to service for adolescent moms…we contacted the people that are in charge and they have distributed the information saying this is what we need to do, and this is what we are going to do...it streamlines that process so that they are not facing those barriers constantly like they were. Now they can get birth control whenever they have the baby, whenever they deliver, they can have it like an implant after they deliver.

Participants explained that through their participation in SCPAF they developed personal relationships with staff at different agencies. When a need was identified, they were able to call their contact directly to coordinate a connection to a resource. In addition, these personal connections allowed them to explain to their EPT clients what to expect when accessing that service.

SCPAF staff tailored their support and services to meet the individual needs of EPT. For example, one participant explained that the SCPAF provided a venue to coordinate resources and determine if they were providing a service that another agency already offered. The participant explained that SCPAF provided a “single point of entry so as not to duplicate services.” Another person explained that SCPAF enabled “smooth handoffs” between agencies to ensure that the needs of EPT were met and that no one fell through the cracks.

However, some partners emphasized that duplication of services is not always a bad thing, especially if duplicated services enhance the accessibility for clients in large rural areas. One partner explained,

We wanted to make sure that we had health care provided in the close vicinity of that client’s residence. If you’re in Jonestown^1^ and Jonestown Medical Center is doing something, that’s not really helping somebody in Rose Hill due to the barrier of lack of transportation. If we have health care partners right up the road, in walking distance, it doesn’t matter that they’re duplicating services, but how accessible is that service to the client? With a county as large as Beech County, you need some duplicating to make sure everybody gets service…. It’s going to take a lot of different partners to reach all of those people.

The presence of SCPAF collaboratives in these communities ensured improved communication among partners. This communication resulted in partners working together to try to make sure services were coordinated and accessible to clients across their communities.

### Partnerships Facilitate Growth in Collaboration Among Partners

Partners across funded communities explained that the collaboration resulting from their participation in SCPAF strengthened their individual organizations. In addition, the SCPAF was integral in developing an infrastructure for enhanced collaboration among those organizations. Prior to the SCPAF grant, most of the partnering agencies were working with the same population of clients without coordinating with each other. For example, partners in one community explained how SCPAF formalized their partnerships with a local fatherhood program:

When this program first started, they were not a part [of it]. To see how that partnership has grown and has become a much more integral part of the process in getting the young dads educated on what it is to be a dad. I have seen the growth of that particular program and the benefits of that program.

Community partners attributed their high level of collaboration to a shared vision and the consistency of partners, but they also credited the SCPAF funds in providing a platform to further leverage those partnerships to address community needs. Because of the SCPAF, partners established a stronger infrastructure for collaboration that included regular meetings. Partners described that the regular partner meetings created a very important opportunity to share and coordinate resources as well as strengthen relationships. The relationships developed or enhanced through the SCPAF partnership, according to partners, made it easier to connect with one another and thereby connect youth with needed resources. One person explained, “I feel like I can call anyone [in this group].” These enhanced personal connections within the collaboratives have resulted in a greater awareness of and therefore connections to resources and services across the community. In addition, connections that have occurred in SCPAF meetings with grant partners have resulted in connecting to other services outside of community collaboratives or identifying further opportunities to collaborate outside of the SCPAF partnerships.

Several communities have partnered on new and additional initiatives because of the sharing and networking fostered through SCPAF. For example, partners in two communities were awarded additional grant funds to address housing needs and to support pregnancy prevention services for teenagers. One community partner explained how its partnership and funding have grown over time: “[It started with] pregnancy prevention community awareness month…then it evolved to the Let’s Talk grant…and then it evolved to the PAF grant and now we’re getting some funds from [another funder]…. The seed has been planted and it’s really grown.”

## Conclusions for Practice

This paper describes stakeholder perceptions about the SCPAF program gathered using REM, an innovative evaluation technique. The SCPAF was designed to support the development of partnerships among youth services agencies in each community to create a system of supportive services for EPT. One salient finding from this evaluation was that stakeholders believe that the support and resources provided by the SCPAF have the potential to change the life course of EPT and their families, including improved education attainment, improved parenting skills, and reduced repeat teen pregnancies. This finding is consistent with prior studies of programs to support expectant and parenting teens (Gruber 2012; Hudgins et al. 2014; Sadler et al. 2007). In addition, participants identified several key successes of the program, including greater accessibility and awareness of support services, reduced duplication of services, and intentional collaboration among partners.

In addition to reporting stakeholder perceptions of the success of SCPAF, this study provides an example of an innovative, participatory evaluation technique. As public health strategies evolve to focus more on community and systems level interventions, evaluation strategies that are adept at capturing information about the successes of community-based collaboratives are needed. As a result, this paper contributes to an expanded body of knowledge regarding evaluation approaches for complex community initiatives. The results identified through this evaluation reflect community stakeholders’ perspectives on what PAF achieved in communities. The REM method synthesized perspectives in real time and allowed participants to elaborate on successes, explain different perspectives, and reflect together to develop a shared narrative.

Program context also has an important impact on implementation of programs and subsequent outcomes (May et al. 2016; Pfadenhauer et al. 2017). REM is adept at capturing critical contextual information on program implementation and the communities in which the SCPAF is embedded. As a result, this paper illustrates how we can align evaluation approaches with intervention approaches that aim to build collaboration. The REM process allows participants to share their own experiences and provide detailed accounts of how the program played out in their own communities. Moreover, although it is a systematic process, the semistructured nature of REM allows the facilitator to pause or veer off course and ask for additional details to ensure that findings are understood within the community context and culture.

More application of the REM technique in varied settings and topics is warranted. The engagement of participants and the utility of information yielded from the evaluation were valuable because REM elucidated the best and most useful parts of the program. REM is an efficient method that gathers a lot of data in a limited amount of time by gathering the perspectives of multiple stakeholders in a single meeting. Therefore, this method may work well for projects with short timelines for data collection. REM would be particularly well suited for community-based programs, collaborative and collective impact–focused initiatives, and policy/systems change projects, given the complex and multifaceted nature of these types of interventions. REM is a valuable method for implementation monitoring and for obtaining stakeholder perspectives of program successes (Chazdon et al. 2017; Kollock et al. 2012; Olfert et al. 2018; Washburn et al. 2018). One potential benefit of the REM technique for a mid-cycle program is that it allows stakeholders the opportunity to synthesize perceived impacts and lessons learned and integrate those into future program planning.

Although this evaluation has many strengths, limitations do exist. A primary limitation is the potential bias in REM participant selection: we could not guarantee that all partners were present at the session. However, a broad range of partners with diverse perspectives was invited to the sessions, and those that did attend the sessions were key stakeholders with knowledge of program implementation. In addition, there is a risk that the REM sessions were implemented inconsistently. To account for this risk, we developed a facilitator’s guide and used it each time to ensure consistent questions and processes. Also, the same team (the facilitator and note taker) facilitated each session. Our use of appreciative inquiry, which focused on the most positive aspects of the program, could be a potential source of bias. However, although our questions did focus on generating ideas about positive aspects of the program, we did not exclude any ideas or perspectives that were negative. That being said, some participants may have felt uncomfortable sharing negative perspectives when the focus was on sharing positive perspectives. Finally, because of the context-specific nature of our findings, the results of these REM sessions cannot be generalized to PAF programs in other communities.

This study has several key strengths. Our evaluation used a participatory approach, wherein we gathered a rich description of stakeholders’ perceptions of the successes of SCPAF. The REM sessions were a valuable tool to engage participants and create energy for further success as they reflected on their past accomplishments. Many stakeholders remarked that they enjoyed the sessions and asked to share the maps and reports with other partners and funders. Moreover, the participatory approach solicited real-time feedback during data collection to ensure interpretations were valid. In addition, we utilized member (or stakeholder) checking as a strategy to enhance the credibility of findings: we provided visuals and reports of our analysis back to session participants to ensure that we accurately represented their perspectives and that no key ideas were absent (Thomas 2006). Finally, the REM technique is a straightforward, efficient, and cost-effective evaluation approach. It generates a lot of information in a short amount of time and is well suited as an evaluation technique to understand complex, collaborative initiatives. Using participatory data collection techniques (such as REM) provided a space for stakeholders to engage in dialogue to synthesize their experiences with SCPAF to construct a shared narrative. We hope that this work will result in enhanced uptake of participatory evaluation techniques to elevate the voices of community members and illuminate multiple perspectives.

## Footnotes

1 Pseudonyms are used for all locations.
